# Correlations between adolescent processing speed and specific spindle frequencies

**DOI:** 10.3389/fnhum.2015.00030

**Published:** 2015-02-09

**Authors:** Rebecca S. Nader, Carlyle T. Smith

**Affiliations:** ^1^Department of Psychology, Trent UniversityPeterborough, ON, Canada; ^2^Department of Psychology, Queen’s UniversityKingston, ON, Canada

**Keywords:** intelligence, processing speed, stage 2, REM, SWS, spindles, sleep

## Abstract

Sleep spindles are waxing and waning thalamocortical oscillations with accepted frequencies of between 11 and 16 Hz and a minimum duration of 0.5 s. Our research has suggested that there is spindle activity in all of the sleep stages, and thus for the present analysis we examined the link between spindle activity (Stage 2, rapid eye movement (REM) and slow wave sleep (SWS)) and waking cognitive abilities in 32 healthy adolescents. After software was used to filter frequencies outside the desired range, slow spindles (11.00–13.50 Hz), fast spindles (13.51–16.00 Hz) and spindle-like activity (16.01–18.50 Hz) were observed in Stage 2, SWS and REM sleep. Our analysis suggests that these specific EEG frequencies were significantly related to processing speed, which is one of the subscales of the intelligence score, in adolescents. The relationship was prominent in SWS and REM sleep. Further, the spindle-like activity (16.01–18.50 Hz) that occurred during SWS was strongly related to processing speed. Results suggest that the ability of adolescents to respond to tasks in an accurate, efficient and timely manner is related to their sleep quality. These findings support earlier research reporting relationships between learning, learning potential and sleep spindle activity in adults and adolescents.

Sleep spindles are an often used hallmark of Stage 2 sleep; these waxing and waning oscillations are commonly observed with frequencies of between 11 and 16 Hz and have durations of between 0.5 and 3 s (Zeitlhofer et al., 1997; DeGennaro and Ferrara, 2003; Schabus et al., 2007; Peters et al., 2008). This frequency range has been further divided by researchers into slow spindles (often between 11 and 13.5 Hz) and fast spindles (often between 13.6 and 16 Hz; Fogel and Smith, 2011). While these are commonly used ranges, there seems to be no clearly defined frequency range for each type (Fogel and Smith, 2011).

It is, however, generally accepted that sleep spindles occur primarily in Stage 2 sleep. They are considered to be markedly reduced in Stage 3 and virtually absent in Stage 4 when EEG records are visually scored (Rechtschaffen and Kales, 1968; Steriade and McCarley, 2005). Steriade and McCarley (2005) have suggested that spindle activity declines as individuals enter deep slow wave sleep (SWS) and only begins to resume when the individual is entering the lighter stages of sleep and preparing for rapid eye movement (REM). Further, the presence of more than a single spindle, without intervening REMs, during an epoch of REM sleep has been considered to be a Stage 2 arousal (Carskadon and Rechtschaffen, 2000).

Spindles have typically been counted visually as opposed to using an automated methodology (DeGennaro and Ferrara, 2003; Ray et al., 2009) and are generally only counted in Stage 2 sleep. Automated spindle counters have become more reliable over the years. One of the advantages of these systems is the ability to filter frequencies that are not of interest, and to allow researchers to visually observe frequencies that are of interest. Ray et al. (2009) validated an automated spindle detection system that modified the settings for each individual subject. By assessing each subject’s average spindle amplitude and setting the minimum amplitude for that subject at 1.96 standard deviations below that mean, the spindle counter was personalized for everyone, allowing for more accurate assessment. Ray et al. (2009) found an overall sensitivity of 98.96% and a specificity of 88.49% using this personalized method.

This automatic spindle counting technique also allows for easy detection of different spindle types (i.e., slow and fast spindles), as well as allowing for the analysis of spindles during different sleep stages. Visual counts of sleep spindles in SWS often lead to the conclusion that there are very few (if any) sleep spindles in SWS. The possibility exists that the sleep spindles are more prominent in SWS than visual inspection would suggest, due to the large amplitude slow waves that make up SWS. Further, examining REM sleep for spindle activity is normally not considered and only two papers actually report a spindle density during REM sleep (Gaillard and Blois, 1981; Zeitlhofer et al., 1997).

Spindle activity in SWS has been observed by other researchers (e.g., Gaillard and Blois, 1981; Zeitlhofer et al., 1997; Steriade and McCarley, 2005; Peter-Derex et al., 2012), but traditionally, spindle activity is assessed primarily in Stage 2 sleep. Using automatic spindle detectors, which can filter out undesired frequencies, researchers can more easily observe the spindle activity that occurs in SWS sleep. Gaillard and Blois (1981) for example, found that sleep spindle activity showed no changes from Stage 2 to Stages 3 and 4. In contrast, Zeitlhofer et al. (1997) found that spindle activity showed a significant decrease from Stage 2 to SWS. Both groups of researchers found that there was spindle activity present in REM sleep, although in both cases it was significantly lower than in Stage 2 and SWS (Gaillard and Blois, 1981; Zeitlhofer et al., 1997).

Sleep spindles do show changes across the lifespan, but the majority of studies have focused on young adults. Nicolas et al. (2001) studied spindle characteristics in individuals from 10 years of age to 69 years of age. Nicolas et al. (2001) found that the number, density, and duration of sleep spindles (in Stage 2) declined with age from early adolescence on. The declines that they observed occurred primarily in the first four decades of life, and Nicolas et al. suggested that these changes are due to a long maturation, rather than aging *per se*. Jenni and Carskadon (2004) examined sigma activity in adolescence, and observed a decrease in the power of the sigma frequency range as adolescents mature from pre- to post-puberty. They also observed a shift in the predominant peak of sigma activity, which they suggest is due to maturation of the thalamocortical system (Jenni and Carskadon, 2004). Tarokh and Carskadon (2010) also found that the peak frequency in the sigma band increased from childhood to early adolescence and that there was a decline in the absolute EEG spectral power across both NREM and REM sleep. Tarokh and Carskadon (2010) suggest that this decline is due to the synaptic pruning which is occurring during adolescence (and beyond). The adolescent period seems to be a time when the brain is engaged in a substantial amount of maturation and the spindle activity that occurs during this period, may not be equivalent to the adult activity.

The current study was designed to investigate two aspects of spindle activity. The first goal was to observe any spindle activity occurring in Stage 2 sleep, as well as SWS and REM in order to determine whether the spindle system really is inhibited during these alternate stages or whether this activity is simply obscured by the other frequencies. The second goal was an attempt to identify whether spindle activity is a marker for cognitive ability or intelligence in children. Some research in adults has supported the idea that certain sleep characteristics such as spindle frequency activity (SFA) and even stage 2 sleep itself, are related to intelligence (e.g., Bódizs et al., 2005; Geiger et al., 2011). Other research has suggested that baseline sleep spindles are related to an individual’s capacity for memory processing and perhaps an inherent learning aptitude or intelligence (e.g., Nader and Smith, 2001, 2003; Schabus et al., 2006; Fogel et al., 2007; Fogel and Smith, 2011). Indeed a number of studies examining sleep spindle development support this idea. Nicolas et al. (2001) for example, observed that spindle activity decreases from adolescence up to the late 60 s and Petit et al. (2004) report that sleep spindles decline with age in terms of their formation, their frequency and their number. It is possible that these declines are related to an age-related decline in cognitive processing. Petit et al. also report that spindle activity declines in patients with dementia, again linking spindle activity with cognition. Another observation that led to the examination of spindles being a possible marker for intelligence/ability is that spindles display significant inter-individual variability but are very consistent within the individual (e.g., Gaillard and Blois, 1981; DeGennaro et al., 2005; Fogel and Smith, 2006).

Research conducted by Nader and Smith (2003) and Fogel et al. (2007) demonstrated that spindle activity was positively correlated with Performance IQ, but not with Verbal IQ (see also, Fogel and Smith, 2011). We predicted that our adolescents would show a similar pattern of results, with spindle activity positively correlated with the more procedural scales of the Wechsler Intelligence Scale for Children (WISC), but with no relationship between the verbal scales of the WISC and spindle activity.

## Method

### Participants

The participants were 32 adolescents (17 female) between the ages of 12 and 19 years (*M* = 15.36 years) recruited from the Peterborough community. Participants were all considered to be healthy and medication free, as assessed by their parents, with no indication of sleep disorders. All subjects were assessed for pubertal development in order to control for hormonal effects on spindle activity.

### Measures

#### EEG recordings

In-home recordings were made using Suzanne™ (Tyco-Healthcare Group LP, Mansfield, MA, USA) portable polysomnographic systems. The sampling rate was 120 Hz and data were stored on PC flash memory cards, and then downloaded off-line onto a PC computer for further analysis. We recorded EEG, electrooculogram (EOG) (horizontal eye movements only), and EMG using silver-plated electrodes. The EEG (C3, C4, FZ, and PZ) and the EOG (right and left eyes) were monopolar recordings and referenced to contralateral electrodes at A1 and A2. The EMG channel was bipolar. For the EEG and EOG channels, the low- and high-pass software filters were set at 0.03 and 30 Hz. For the EMG channel, only frequencies above 10 Hz were recorded.

Sleep stages were generally scored according to standard criteria (Rechtschaffen and Kales, 1968). However, we sometimes deviated slightly from traditional protocol when scoring the REM sleep stage. The appearance of spindles during REM sleep in the raw EEG was rare, and they only became more visible in the filtered channel. However, according to standard criteria, the observation of a spindle would normally signal an ending to the REM period and the beginning of a period of Stage 2 with the appearance of other Stage 2 indicators. It would also be expected that there would be some increased activity in the EMG channel. If there was absolutely no change in the EMG, no other sign of a Stage 2 intrusion (such as a K-complex) and further REM bursts, the epoch was counted as REM sleep despite the appearance of a spindle. Sleep spindles were counted using the automated spindle counter PRANA® (PhiTools, Strasbourg, France). For each spindle type, an expert technologist identified and recorded the peak amplitudes of 15 spindles in each of the first and second halves of the night for Stage 2 (30 spindles in total for each spindle type). Values were then used to calculate the mean and standard deviation of peak amplitude for each subject. The minimal amplitude criterion for the automated spindle counter was determined by subtracting 1.96 SD units from each mean. This procedure was repeated for each subject. Included in the study were spindle-like waves in the 16–18.50 Hz range. We will use the term “spindle-like” rather than spindle throughout. While these waves share many characteristics of the spindle, their frequencies are in the 16.01–18.50 Hz range. This EEG activity appears to varying degrees in all individuals (Nader et al., 2012a,b,c). The same minimum spindle amplitudes were used in each of the sleep stages (Stage 2, SWS, and REM).

Intelligence was assessed using the Wechsler Intelligence Scale for Children- Fourth Edition (WISC-IV) Canadian Edition. Tests were administered individually by a registered psychometrist. Five participants were assessed by the same psychometrist using the Wechsler Adult Intelligence Scale—Third Edition (WAIS-III) as they were above the age for the WISC-IV.

A number of correlations were performed on sleep spindle activity as it relates to age and IQ in the different sleep stages. Since the measures examined were of the ratio order (spindle density, age) and interval order (IQ scores), we utilized the Pearson correlation. Despite the possible non-normality of some of the data, the non-parametric Spearman’s correlation was rejected because reduction of the data to an ordinal level would result in considerable loss of information and power. The Pearson is known to be quite robust, even with non-normal distributions and with our relatively small sample size, was considered the most appropriate.

All subjects were assessed for pubertal development, using the Tanner Scale, in order to control for hormonal effects on spindle activity.

This study was approved by the Trent University Research Ethics Board.

## Results

**Figure 1:**
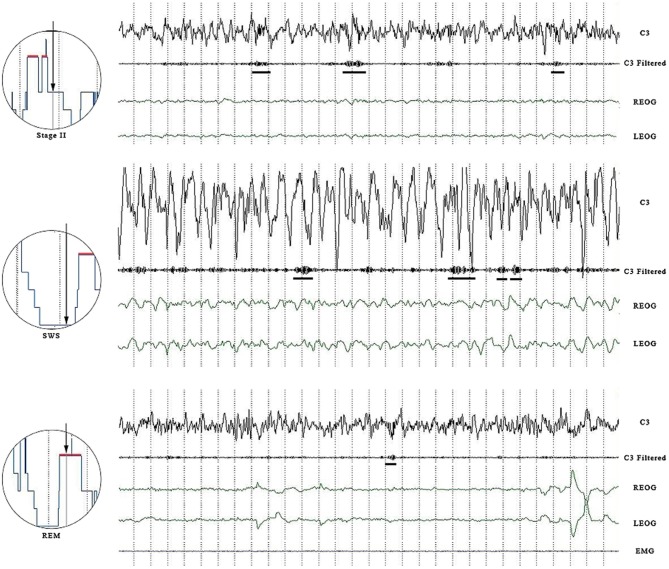
**Panel shows an epoch of SWS, Stage 2 and REM sleep**. Channels are C3 raw, C3 filtered (11.00–16.0 Hz), left and right EOG. For the REM panel, EMG is also included. Both slow and fast spindles are displayed. Horizontal bars underline wave bursts counted as spindles.

Sleep spindles were detected in all sleep stages (see Table 1 for densities), not just in Stage 2. Spindle counts varied among the different stages of sleep, with Stage 2 having the highest density and REM having the lowest density. Despite substantial variability (particularly in REM where one individual may have exhibited no spindles at a specific electrode site while another may have exhibited over 100 spindles), a significant number of our adolescents showed spindle activity during REM. In fact, the number of individuals showing more than 30 spindles during REM sleep was substantial, with eight individuals (25%) displaying more than 30 slow spindles and six individuals (19%) showing more than 30 fast spindles during REM sleep. From the visual EEG, it was clear that these young healthy subjects were not exhibiting Stage 2 intrusions into REM sleep during the night (see Figure 1).

**Table 1:** **Mean density (spindles/minute) and mean number (±SD) of sleep spindles or spindle-like activity in Stage 2, SWS and REM**.

	Number								
	11.00–13.5 Hz			13.51–16 Hz			16.01–18.5 Hz		
	Stage 2	SWS	REM	Stage 2	SWS	REM	Stage 2	SWS	REM
C3	1656.36	793.68	21.69	348.97	104.34	12.55	36.16	17.06	10.74
	(519.84)	(604.42)	(34.38)	(291.76)	(104.95)	(17.12)	(41.12)	(33.46)	(20.56)
C4	1557.19	688.28	19.25	442.41	139.09	16.25	40.41	20.59	12.19
	(545.73)	(535.08)	(29.03)	(368.60)	(107.94)	(32.36)	(46.47)	(35.58)	(23.63)
FZ	1776.09	1019.88	19.63	365.66	126.63	19.06	42.31	12.28	18.13
	(448.50)	(624.16)	(18.85)	(280.72)	(88.21)	(32.44)	(54.49)	(19.10)	(33.38)
PZ	1568.94	646.97	15.71	437.94	142.61	5.16	19.26	11.68	4.03
	(468.79)	(540.44)	(23.72)	(362.27)	(155.82)	(5.96)	(24.17)	(20.13)	(6.90)
	**Density**								
	**11–13.51 Hz**			**13.51–16 Hz**			**16.01–18.5 Hz**		
	**Stage 2**	**SWS**	**REM**	**Stage 2**	**SWS**	**REM**	**Stage 2**	**SWS**	**REM**
C3	6.65	5.86	0.18	1.38	0.77	0.11	0.14	0.11	0.10
	(2.22)	(3.77)	(0.28)	(1.09)	(0.63)	(0.14)	(0.16)	(0.18)	(0.19)
C4	6.21	5.05	0.15	1.74	1.08	0.13	0.15	0.14	0.11
	(2.10)	(2.94)	(0.21)	(1.35)	(0.78)	(0.21)	(0.18)	(0.20)	(0.21)
FZ	7.14	7.54	0.17	1.45	0.97	0.18	0.16	0.08	0.18
	(1.87)	(3.77)	(0.16)	(1.05)	(0.55)	(0.33)	(0.18)	(0.11)	(0.34)
PZ	6.24	4.74	0.12	1.73	1.05	0.05	0.07	0.08	0.04
	(1.82)	(3.19)	(0.15)	(1.38)	(0.98)	(0.06)	(0.09)	(0.11)	(0.07)

Interestingly, the 16.01–18.50 Hz spindle—like activity was also observed in all subjects in every phase of the sleep night. While the number of these incidents was less than that for conventional spindle activity, the relative frequency of occurrence in each sleep stage and density of this event showed several similarities (Table 1). For example, the 16.01–18.50 Hz activity showed a similar pattern to the slow spindle (11.00–13.50 Hz) activity, with the greatest number appearing in the frontal region and fewer in the parietal region. This is in contrast to the fast spindle (13.51–16.00 Hz) activity, which was most prominent in the parietal region.

We do not think these waves are artifacts for several reasons. During scoring, all epochs with movement artifacts were discarded. They do not appear in any time locked form that we can see in relation to the other spindle types. Thus we do not think they are some kind of “echo” of the other spindles. Our system is capable of separating these frequency bands such that we do not think they are scoring errors related to spillover activity from spindles in the 13.50–16.00 Hz range. These 16.01–18.50 Hz waves appear to occur on their own time, unrelated to the other two spindle types and can even be seen to occur simultaneously on occasion, suggesting that they are governed by an independent generator. They are more prevalent at the Fz derivation, although they are present at C3, C4 and Pz as well, suggesting their origin is more frontal. They also appear to be smaller in size than spindles measured between 11.00 and 16.00 Hz. There were differences in the amplitudes of the three spindle types in Stage 2. At Fz, for example, the 16.01–18.50 Hz waveforms have significantly smaller average amplitudes (34.27 ± 8.21 uV) than slow (43.95 ± 8.73 uV) or fast (44.84 ± 7.39 uV) spindles which do not differ [*F*_(2,6)_= 45.04, *p* < 0.000001]. They also have quite different densities (spindles/minute) as can be seen from the Table 1. The three frequency ranges were found to have significantly different densities (spindles/minute), with slow spindles being most prevalent (7.14 ± 1.87), then fast spindles (1.45 ± 1.05) and finally the 16.01–18.50 Hz range (0.16 ± 0.18), [*F*_(2,60)_ = 300.20, *p* < 0.000001].

The three frequency ranges were also compared for mean duration in Stage 2 sleep. An ANOVA showed that there was a significant main effect of frequency range, *F*_(2,62)_ = 345.415, *p* < 0.000001. Slow spindles (*M* = 1.74 s) had significantly longer durations than fast spindles (*M* = 1.381 s) and the 16.01–18.50 Hz range (*M* = 0.892 s). Fast spindles also had a significantly longer duration than the 16.01–18.50 Hz waveform. All of these factors lead us to believe that the 16.01–18.50 Hz activity is a separate waveform worthy of further investigation.

### Sleep spindle activity and age

Sleep spindle density was correlated with age in order to determine whether the appearance of spindles in the various stages varied with age. The density of slow spindles in Stage 2 sleep was negatively correlated with age in the frontal region (FZ; *r*_(30)_ = −0.37, *p* < 0.05). The density of fast spindles at C4 in Stage 2 was positively correlated with age (*r*_(30)_ = 0.35, *p* < 0.05). This suggests that there may be a tendency for the density of slow spindles in Stage 2 to decline with age and a tendency for the density of fast spindles to increase with age across adolescence. There were no other significant correlations with age, suggesting that the spindle densities in SWS and REM are not strongly related to the age range in this adolescent group. It also suggests that age may not be a factor in the appearance of the activity in the 16.01–18.50 Hz range.

We also examined the relationships between age and spindle duration in Stage 2 sleep. Correlations showed that the duration of the slow spindles showed a significant decline with age in three of our four derivations [C3: *r*_(30)_ = −0.38, *p* < 0.05; C4: *r*_(30)_ = −0.48, *p* < 0.01; FZ: *r*_(30)_ = −0.50, *p* < 0.005]. Spindle amplitude showed a similar pattern of results, slow spindle amplitude declined at C4 (*r*_(30)_ = −0.48, *p* < 0.01), Fast spindle amplitude declined significantly at all four of our electrode locations [C3: *r*_(30)_ = −0.41, *p* < 0.05; C4: *r*_(30)_ = −0.56, *p* < 0.001; FZ: *r*_(30)_ = −0.36, *p* < 0.05; PZ: *r*_(30)_ = −0.49, *p* < 0.01] in Stage 2 sleep. The 16.01–18.50 Hz waveform showed a similar trend toward an age related decline in Stage 2 sleep, but was only significant at C4 (*r*_(30)_ = −0.39, *p* < 0.05). Taken together, these results suggest that there is an age related decline in spindle amplitude for all frequencies during the adolescent period.

Similar to age, there were no significant correlations between pubertal development (Tanner Stages) and spindle density (11.00–13.50 Hz and 13.51–16.00 Hz) in Stage 2, SWS and REM. However, when the activity in the 16.01–18.50 Hz range was correlated with the Tanner stages, there were some significant relationships observed. The spindle density in this frequency range was found to be significantly, positively related to pubertal development in Stage 2 in C3 (*r*_(27)_ = 0.39, *p* < 0.05), C4 (*r*_(28)_ = 0.42, *p* < 0.05) and PZ (*r*_(27)_ = 0.42, *p* < 0.05) and to show a trend toward a positive relationship in FZ (*r*_(28)_ = 0.34, *p* < 0.10).

A similar pattern was observed in SWS, where the density of activity in the 16–18.5 Hz range was significantly, positively related to Tanner Stage. This positive relationship was observed in C4 (*r*_(28)_ = 0.39, *p* < 0.05) and PZ (*r*_(27)_ = 0.40, *p* < 0.05) and a trend toward this positive relationship was observed in C3 (*r*_(27)_ = 0.33, *p* < 0.10). The activity in REM sleep did not vary with pubertal development as measured by the Tanner Stages.

### Spindle activity and IQ

Spindle activity in Stage 2, SWS and REM was correlated with the Full Scale IQ from the WISC-IV. We did not expect to see any significant correlations between spindle density and any of the Verbal subscales (e.g., verbal comprehension), although we did expect that there would be significant correlations with Picture Completion (perceptual organization) and Processing Speed (see Fogel and Smith, 2011). Consequently, we confined our correlations to Full Scale IQ and these two procedural traits. There was only one significant correlation between the EEG activity and Full Scale IQ (in SWS, density of 13.5–16.00 Hz activity was negatively related to Full Scale IQ; *r*_(32)_ = −0.351, *p* < 0.05).

However, a pattern of significant correlations emerged when the procedural IQ subscales were examined. Since Age was observed to be positively related to both Processing Speed and percentage of SWS, partial correlations, controlling for age, were conducted between these subscales and the various EEG frequencies. Processing speed appeared to be highly related to Spindle Density, particularly during REM and SWS. Table 2 presents the pattern of significant correlations between Processing Speed and EEG activity. An estimated total of 108 Pearson correlations were run [Derivation (4), Spindle Type (3), Sleep Stage (3)]. While this could be considered to be a large number of correlations requiring some kind of correction for Type I Error, we did not do so for several reasons. Our sample size was quite small and thus applying such corrections as Bonferroni would have been too conservative. Further, the spindle types, EEG derivations and sleep states are undoubtedly not completely independent of each other and this reduces the need for correction. Also, the consistent patterns in the results suggest that these findings are not random and do warrant further examination. While our predictions were partially confirmed, this is an exploratory study and the data provide new research directions.

**Table 2:** **Partial correlations (controlling for Age) between spindle densities (spindles/minute) and processing speed, organized by frequency and sleep stage**.

	Stage 2	SWS	REM
C3 11.00–13.5 Hz	0.47*	0.09	0.42*
C3 13.5–16 Hz	−0.04	−0.01	0.43*
FZ 13.5–16 Hz	0.03	0.01	0.53*
C3 16–18.5 Hz	0.33	0.45*	0.19
C4 16–18.5 Hz	0.26	0.32*	0.17
FZ 16–18.5 Hz	0.27	0.37*	0.31
PZ 16–18.5 Hz	0.22	0.46*	0.08

*p < 0.05.

As the bulk of the significant correlations seemed to be between EEG activity in REM and SWS, with only a single significant correlation in Stage 2, a regression analysis was conducted using SWS and REM activity. A regression analysis was performed on Processing speed, with age being entered first to control for its effects (*R* = 0.484, *p* < 0.01). Proportion of SWS and proportion of REM sleep were entered into the equation next (*R* = 0.654, *p* < 0.01). Finally the variables C3 (11.00–13.50 Hz) REM, C3 (13.5–16.00 Hz) REM, FZ (13.5–16.00 Hz) REM, C3 (16–18.50 Hz) SWS, FZ (16–18.50 Hz) SWS, PZ (16–18.50 Hz) SWS were entered into the equation (*R* = 0.803, *p* < 0.01). The regression analysis suggests that the measures of age and sleep EEG account for 64.6% (adj. *R*^2^ = 0.486) of the variance in processing speed. While it is not surprising that age contributes a large proportion of the variance, the results underline the importance of the activity in REM and SWS rather than Stage 2.

## Discussion

Spindles are not limited to Stage 2 sleep and appear in all the sleep stages of healthy adolescents. It is possible that the appearance of spindles in SWS and REM could be due to a developmental process of adolescence, but correlations of spindle density with age and puberty showed few consistent significant relationships, with the exception of a positive relationship between density of the faster wavelengths in SWS and Tanner stage. This suggests that the appearance of spindles in REM and SWS may not be a consequence of development, and instead may be a consistent robust phenomenon. It is possible that the faster wavelengths increase in density with pubertal development. As the brain undergoes its substantial maturation during adolescence, it may become more physically able to produce these faster wavelengths during SWS.

Our results do suggest that, despite the lack of consistent changes in density, other measures of spindle activity may be changing across the adolescent age range. Slow spindle duration declines over adolescence in Stage 2, while there is no change in the duration of the fast and 16.01–18.5 Hz waveforms. Amplitude declined in all three frequency bands across adolescence in Stage 2. These results seem to be in agreement with those of Nicolas et al. (2001), Jenni and Carskadon (2004) and Tarokh and Carskadon (2010).

The density of spindles during SWS is significantly less than the spindle density observed during Stage 2, but it is still quite substantial (see Figure 1). Our results of a decreased density in SWS is consistent with the findings of both Zeitlhofer et al. (1997) and Andrillon et al. (2011) who observed a significant decrease in spindle density from Stage 2 to Stage 3 to Stage 4. Despite a difference in the electrodes used to measure EEG (Andrillon et al. used depth electrodes), the results from the present study are in agreement with the findings of Andrillon et al. (2011) who observed more slow spindles in the frontal region than in the parietal region and more fast spindles in the parietal region than in the frontal region.

The density of sleep spindles in REM is low, but certainly not completely absent. Our data corresponds very closely to data reported by Gaillard and Blois (1981) who examined spindle activity in adults. Using a filter system, which isolated frequencies between 11.6 and 17.2 Hz, Gaillard and Blois found spindle densities in REM sleep that were very similar to the results presented here. These researchers found a spindle density of 0.87 spindles per minute (±1.74), supporting the idea that while there is great variability in the number of spindles that occur during REM, they are certainly not absent during this stage of sleep. Our results are also similar to those found by Zeitlhofer et al. (1997), although our spindle densities in REM (*M* = 0.18 at C3) were lower than their findings (*M* = 1.3 spindles/minute).

The appearance of spindles in REM suggests that the mechanism that produces sleep spindles is not completely inhibited or absent during REM. It is possible that the separate phasic systems that produce eye movements and spindle activity cannot occur simultaneously, but apparently they can occur in close succession. While it has previously been accepted that the appearance of sleep spindles in REM is actually a Stage 2 intrusion (Carskadon and Rechtschaffen, 2000), it seems unlikely that one-quarter of our healthy, young subjects would have more than 30 Stage 2 intrusions during the REM period. In fact, our visual scoring procedure revealed no spindles and certainly no Stage 2 intrusions (Carskadon and Rechtschaffen, 2000). It was only when we filtered out the other frequencies that we were able to count the spindles occurring in REM sleep. The spindles in REM sleep met the same criteria for amplitude and duration, as did the spindles in Stage 2 in order to be counted.

The data suggest that, with the advent of more sophisticated measuring techniques, spindles that occur during SWS and REM are phenomena that have been mostly overlooked, because they were not easily observable. In future, it would be valuable to include all of the sleep stages as well as to examine possible sleep spindle activity in frequency ranges from 11–19 Hz.

While spindle density, in any frequency range, was not very strongly related to age (at least within our adolescent age group), it does appear that pubertal development plays a role in the appearance of some of these spindles. An increase in density of the activity in the 16.01–18.50 Hz range was observed in conjunction with an increase in pubertal development in both Stage 2 and SWS. It is possible that this activity is related to maturity and brain development and may be involved in the establishment of higher order cognitive abilities. A tentative hypothesis is that the link between IQ measures and sleep states develops over the adolescent period, as the brain matures to its adult state.

The sleep measures were correlated with Full Scale IQ, and its subscales to try and assess whether there are any biological markers for intelligence in adolescents. Research in adults has suggested that sleep spindle activity may be linked with an aptitude for learning (Nader and Smith, 2001; Schabus et al., 2006; Fogel et al., 2007; Fogel and Smith, 2011). Given the substantial development in the brain that occurs over the adolescent period, we were interested in whether there was any support for this relationship in adolescents. Dang-Vu et al. (2010) observed that the faster spindle activity was associated with more extensive cortical activation; the results from our adolescents suggest that it is the faster (or higher frequency) brain activity that is associated with some forms of intelligence.

Using the WISC-IV (or WAIS-III), Full scale IQ and the procedural subscales were correlated with brain wave activity in three frequency ranges. The first two ranges, 11.00–13.50 Hz, and 13.5–16.00 Hz, are traditional spindle frequencies; the third frequency range (16–18.50 Hz) is above the normal spindle range, but we observed consistent activity in these frequencies in the current sample and in an earlier study (Nader and Smith, 2001) and felt it was important to include this activity in our examination. While Full Scale IQ was not related to any of the measured brain activity, some of the subscales were. Processing Speed in particular, seemed to be strongly related to the brain wave activity during sleep. Processing speed is a skill that is linked to executive functioning (Jacobson et al., 2011) and requires individuals to be able to complete a task accurately and as quickly as possible. Processing Speed was observed to be positively associated with age in this adolescent group; this positive association may be due to the development of executive functioning that occurs in adolescence. Executive functioning involves the ability to plan, coordinate, and execute behavior (Blakemore and Choudhury, 2006), processing speed requires the individual to not only perform at a rapid pace, but to be able to respond both efficiently and accurately (Jacobson et al., 2011). This ability requires the individual to plan and prepare for stimulus orientation and appropriate responses (Jacobson et al., 2011).

Performance on the Processing Speed task was observed to be positively related to age, the proportion of both SWS and REM sleep, and the EEG activity that occurs during these stages. Due to these observed relationships, a regression analysis was run to determine how much variance in Processing Speed scores could be accounted for by these sleep variables. Using this exploratory analysis, we were able to account for 64.5% of the variance in Processing Speed by knowing age, proportion of REM and SWS, the density of activity in the 16.00–18.50 Hz range during SWS and the density of activity in the 11.00–13.50 Hz, and 13.51–16.00 Hz ranges during REM. This suggests that the ability of adolescents to respond in an efficient and accurate manner to the task at hand is related to sleep quality. In fact, it may be that the spindle activity during REM and SWS is indicative of their Processing Speed abilities. Since we were not able to predict Full Scale IQ, these results suggest that only specific components of intelligence are related to sleep state activity. This supports research conducted with adolescents and adults, which has suggested that some measures of IQ are related to sleep and also that sleep spindles are related to how well we learn (e.g., Nader and Smith, 2003; Schabus et al., 2006; Fogel et al., 2007).

There are some things that should be considered in future studies. As this was an exploratory study with a limited number of participants, further research needs to be done to confirm the findings presented here. The small number of participants did limit power, and we did not apply any correction procedures for Type I error. However, we did limit the number of correlations performed and only examined the relationships between spindle activity and the Performance IQ/Procedural tasks, along with Full Scale IQ. Further research would be able to use a larger sample and correct for Type I error. Also, the scoring system (Ray et al., 2009) was developed using the EEG from young adults and validating it for younger participants would be valuable. It is possible that the number of false positives might have been different in our younger participants. We can only say that there was no consistent increase in the spindle count as we looked at younger subjects. Depending on spindle type, some were positively correlated with age while some were negatively correlated with age or not correlated at all. This suggests that there was no general increase in false positives.

## Conflict of interest statement

The authors declare that the research was conducted in the absence of any commercial or financial relationships that could be construed as a potential conflict of interest.
